# A Quantitative and Qualitative Review on the Main Research Streams Regarding Blockchain Technology in Healthcare

**DOI:** 10.3390/healthcare9030247

**Published:** 2021-03-01

**Authors:** Yong Sauk Hau, Min Cheol Chang

**Affiliations:** 1 Department of Business Administration, School of Business, Yeungnam University, Gyeongsan 38541, Korea; augustine@yu.ac.kr; 2 Medical Management Research Center, School of Business, Yeungnam University, Gyeongsan 38541, Korea; 3 Department of Physical Medicine and Rehabilitation, College of Medicine, Yeungnam University, Taegu 38541, Korea

**Keywords:** blockchain, healthcare, review, electronic medical record, cloud, internet of things, technology convergence

## Abstract

(1) Background: Blockchain technology has been gaining high popularity in the healthcare domain. This has brought about a spate of recent studies regarding blockchain technology in healthcare, creating high demand for quantitative or qualitative reviews on the main research streams thereof. In order to contribute to satisfying the high demand, this research presents a quantitative and qualitative review on studies regarding blockchain technology in healthcare. (2) Methods: A quantitative review was performed by searching the Web of Science database for articles published until 10 March in 2020, and a qualitative review was conducted by using the content analysis based on the integrative view of Leavitt’s diamond model. (3) Results: The quantitative review identified five research streams. The number of articles about blockchain technology in healthcare has dramatically increased since 2016, with a compound annual growth rate of 254.4%. English is the most dominant language used in the articles, and the USA and China are the top two countries of origin of the articles, representing overwhelming portions. The IEEE Access, Journal of Medical Systems, Journal of Medical Internet Research, Applied Sciences Basel, and Sensors are the top five journals in terms of publication. The articles showed an L-shaped distribution in terms of their annual average numbers of citations. The qualitative review revealed two research streams. Most of the top 10 articles ranked by their annual average numbers of citations concentrated on developing or proposing new technological solutions using blockchain technology to effectively revolutionize the current methods of managing data in the healthcare domain. The majority of the top 10 articles pursued the convergence of blockchain technology with cloud technology or IoT. (4) Conclusions: This article illuminates the main research streams about blockchain technology in healthcare through a quantitative and qualitative review, providing implications for future research on blockchain technology.

## 1. Introduction

Blockchain technology, a distributed ledger based on peer to peer networks [1], has been gaining high popularity in healthcare [2,3]. This high popularity has resulted from the innovative advantages of blockchain technology in managing medical data when compared to conventional methods. For example, blockchain technology can enhance not only the security of patients’ medical data in hospitals [4,5], but also the safety of their medical data transfer between hospitals [6,7]. It can ensure that patients have unrestricted access to their own medical data whenever and wherever they require [6].

Furthermore, blockchain technology can provide innovative medical services for both patients and healthcare organizations through technological convergence with cutting edge information technology (IT) such as cloud technology [7,8], internet of things (IoT) [9,10], big data [11], and smart devices [10]. Therefore, more and more researchers and practitioners in healthcare are paying special attention to blockchain technology. This interest has recently brought about many studies on blockchain technology in healthcare, creating high demand for quantitative or qualitative reviews on the main research streams thereof. In order to contribute to satisfying this high demand for the reviews, this research conducts not only a quantitative review but also a qualitative review on studies about blockchain technology in healthcare. For the quantitative review, this research analyzes the main research streams in terms of their distribution by publication year, language, country of origin, journal, and the annual average numbers of citations. For the qualitative review, this research examines the contents of the top ten studies ranked by their annual average numbers of citations through the lens of Leavitt’s diamond model [12]. Leavitt’s diamond model [12] is a widely used theoretical framework for analyzing the impact of new technology on an organization by considering its impact on the inter-relationships between the four major factors of the organization—technology, people, structure, and task—in an integrative view.

In this article, we present a quantitative and qualitative review on prior studies about blockchain technology in healthcare and illuminate the main research streams thereof with a view to providing useful implications for future research.

## 2. Methods

**Figure 1:**
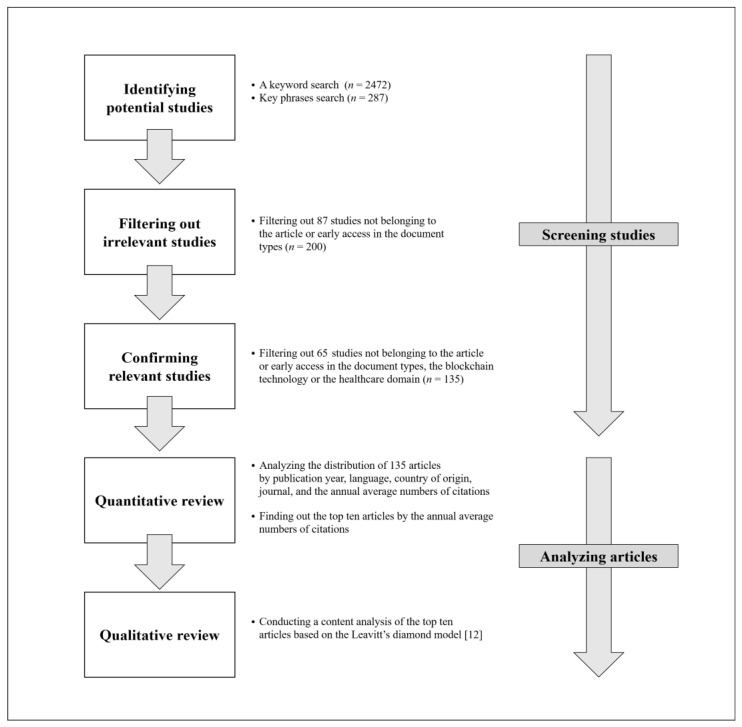
Review flow chart.

This study conducts a quantitative and qualitative review to effectively illuminate the main research streams regarding blockchain technology in healthcare. Our quantitative review aims at not only revealing the main research streams in terms of the distribution of relevant studies by their publication year, language, country of origin, journal, and the annual average numbers of citations, but also identifying the top ten articles ranked by their annual average numbers of citations. Our qualitative review focuses on analyzing the main content trends in the top ten articles using the solid theoretical basis of Leavitt’s diamond model [12]. The following two subsections describe the quantitative and qualitative methods used for this review, and Figure 1 summarizes our review flow.

### 2.1. Quantitative Method

We searched the Web of Science database for studies published until 10 March in 2020. Studies on blockchain technology in healthcare were observed to be published not only in medical journals but also in other journals from various domains, including IT, law, engineering, economics, and business administration. Therefore, we adopted the Web of Science database as the source of articles for this review, considering its extensive coverage of a variety of journals in both the natural and social sciences.

In order to perform a rigorous quantitative review, we used four steps, including identifying potential studies, filtering out irrelevant studies, confirming relevant studies, and analyzing selected articles.

In the first step of identifying potential studies, we performed an initial search for studies containing a keyword such as “blockchain” in their titles, abstracts, or keywords. This step identified a total number of 2472 potential studies. In another search, we narrowed down these studies to those containing key phrases, such as “blockchain” combined with “medic~”, “health~”, “biomedi~”, “clinic~”, or “hospital~” in their titles, abstracts, or keywords. This yielded a total number of 287 potential studies.

In the second step of filtering out irrelevant studies in terms of the document types, the 287 potential studies were filtered into a total number of 200 articles which belonged to the article or early access in the document types classified by the Web of Science database. According to this database, the early access indicated articles published online ahead of official publication.

In the third step of confirming the relevancy of the 200 remaining studies by their document types and contents, we examined their document types, titles, abstracts, and keywords and filtered out 65 studies that satisfied the criterion of our search with the key phrases but did not belong to the article or early access in the document types, the blockchain technology or the healthcare domain. This step confirmed a total number of 135 articles, including the early access, which were used for our review.

In the fourth step of analyzing the 135 articles, we investigated their distribution by publication year, language, country of origin, journal, and the annual average numbers of citations. The countries of origin of the articles were examined by identifying the nationalities of the organizations to which the authors belonged. The country of origin of an article was evaluated to be two countries if the article had two authors working for organizations in two different countries. The annual average numbers of citations of the 135 articles were used to identify the top ten articles. The annual average numbers of citations can more effectively show the degree of influence of each article on other research than the total number of citations, by controlling the impact of time after publication. For example, an article published in 2016 is likely to have been cited more frequently than articles published in 2019. Therefore, we used the annual average numbers of citations to determine the ranks of the 135 articles.

### 2.2. Qualitative Method

**Figure 2:**
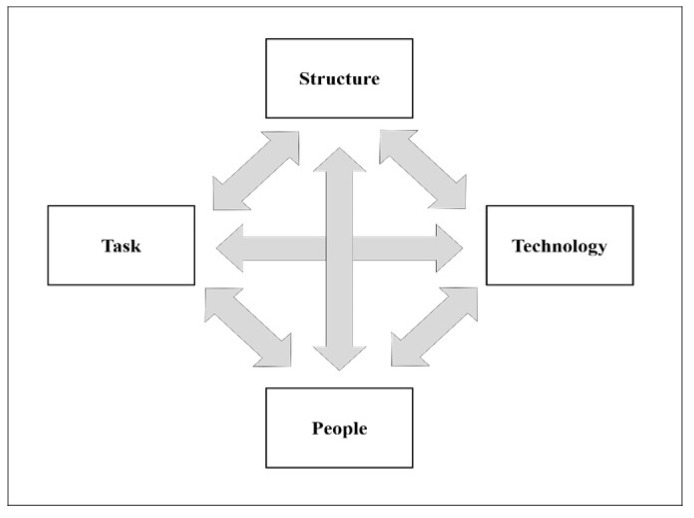
Leavitt’s diamond model [12] (note: the source of this figure is page 1145 of [12]).

We conducted a content analysis of the top ten articles under the integrative view of Leavitt’s diamond model [12]. Leavitt’s diamond model [12] is well-known for its integrative perspective of the influence of new technology on an organization, based on the inter-relationships between the four factors of an organization such as structure, task, technology, and people, as illustrated in Figure 2. The diamond model [12] has been widely used to analyze the impact of new technology on organizations since it was introduced by Harold J. Leavitt, a psychologist in organizational behavior in the field of management.

According to Leavitt’s diamond model [12] in Figure 2, in the healthcare domain, the structure is the organizational hierarchy or the subsystem of work process and communication in healthcare organizations. The task refers to the work necessary to produce healthcare services or goods [12]. The technology indicates the IT programs or resources to support the processes of healthcare [12]. Finally, the people include the medical or administrative staff in healthcare organizations [12]. Leavitt’s diamond model [12] considers healthcare organizations as complex systems wherein their structure, task, technology, and people have significant interactions with one another. This interaction can mean that the application of blockchain technology to healthcare organizations influences not only their technology but also other factors such as structure, task, and people in these organizations [12]. According to Leavitt [12], major approaches applied to the change in healthcare organizations on the adoption of blockchain technology can be classified into three approaches by using three of the four factors in the diamond model: the people approach, the structural approach, or the technological approach. Therefore, this study performs a content analysis of the top ten articles under the integrative perspective of Leavitt’s diamond model [12] by examining which approach, among the people, structural, and technological approaches, has been adopted in the articles to reveal the main research streams therein.

The content analysis was performed by the two authors of this article, who are experts in IT and medicine, respectively. One author has a Ph.D. in IT management, and the other is a professor in the School of Medicine. We examined which approach, among the people, structural, and technological approaches according to Leavitt [12], was adopted in the contents of the top ten articles to analyze the main streams therein.

## 3. Results

The following two subsections report our review results according to the two review methods used in this study. The first subsection presents the results using the quantitative method, and the second subsection provides the results using the qualitative method.

### 3.1. Quantitative Review Results

Our quantitative review revealed five major research streams in the 135 articles about blockchain technology in healthcare in terms of their distribution by publication year, language, country of origin, journal, and the annual average numbers of citations as follows.

**Figure 3:**
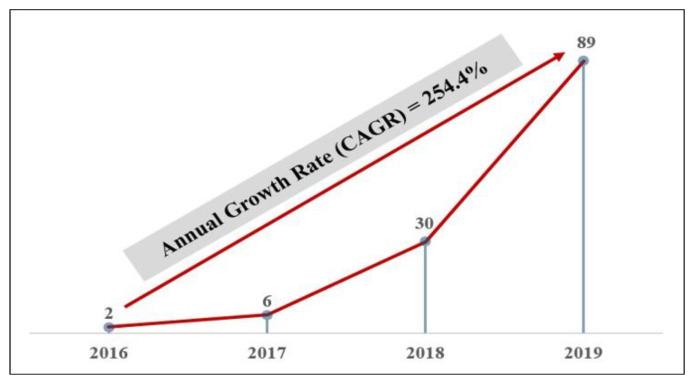
Distribution of the articles by publication year (2016~2019) (note: CAGR: Compound Annual Growth Rate; As of 10 March in 2020, 8 articles were published in 2020 but they were not included in this figure).

First, the number of articles has dramatically increased since 2016. These articles began being published in 2016. In 2016, only two articles were published. Since then, however, the number of published articles has grown rapidly, with a high compound annual growth rate of 254.4%, as shown in Figure 3. In greater detail, 6 and 30 articles were published in 2017 and 2018, respectively. In 2019, 89 articles were published, further showing the rapid growth in publication volume.

**Figure 4:**
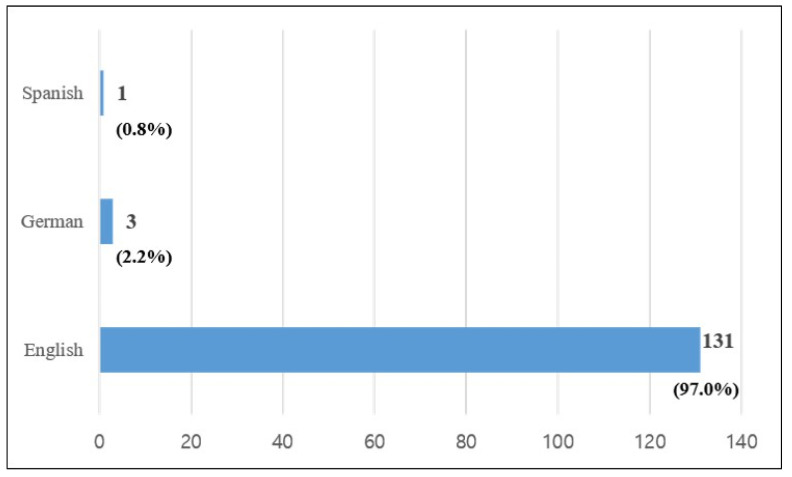
Distribution of the articles by language.

Second, English is the most dominant language used across the 135 articles. A total of 131 articles were written in English (97%). The other four articles included three in German (2.2%) and one (0.8%) in Spanish, as summarized in Figure 4.

**Figure 5:**
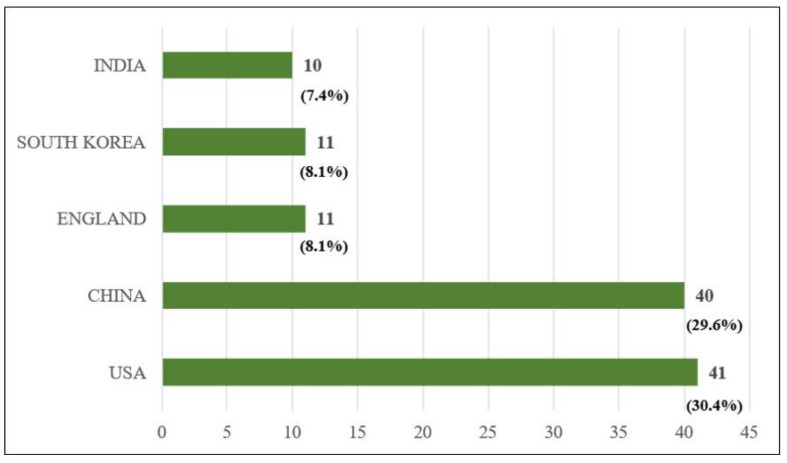
Top five countries of origin.

Third, the USA, China, England, South Korea, and India are the top five countries of origin. A total of 41 articles could be traced to researchers in the USA (30.4%), 40 from China (29.6%), 11 from England (8.1%), 11 from South Korea (8.1%), and 10 from India (7.4%), as shown in Figure 5. Appendix A reports the distribution by country of origin for all 135 articles.

**Figure 6:**
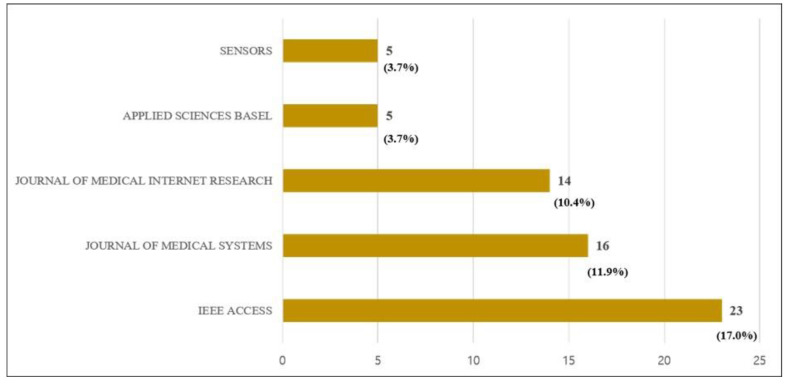
Top five journals in the 135 articles in terms of publication.

Fourth, the IEEE Access, Journal of Medical Systems, Journal of Medical Internet Research, Applied Sciences Basel, and Sensors are the top five journals in terms of their share among 61 journals in which the 135 articles were published. A total of 23 articles were published in the IEEE Access (17.0%), 16 articles in the Journal of Medical Systems (11.9%), 14 articles in the Journal of Medical Internet Research (10.4%), 5 articles in the Applied Sciences Basel (3.7%), and 5 articles in the Sensors (3.7%), as summarized in Figure 6. Appendix B reports the specific distribution of all journals.

**Figure 7:**
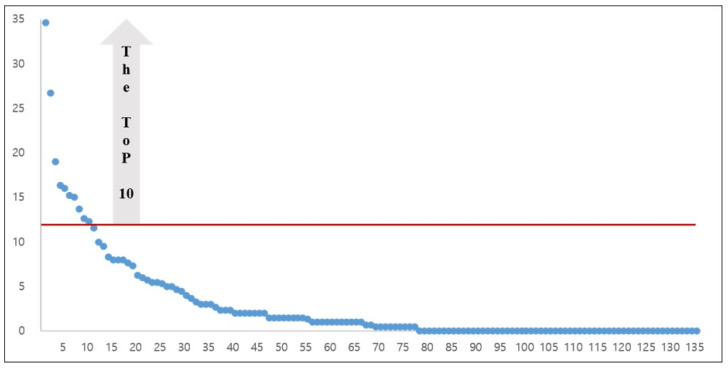
Distribution of the articles by the annual average numbers of citations (note: The blue dots indicate each of the 135 articles; *X*-axis shows the rank of the 135 articles by the annual average numbers of citations; *Y*-axis indicates their annual average numbers of citations; The blue dots above the red line are the top ten articles ranked by their annual average numbers of citations).

Fifth, the 135 articles show an L-shaped distribution as illustrated in Figure 7, arranged by their ranks in terms of the annual average number of citations. The minimum, median, and maximum values of the L-shaped distribution were 0, 0.67, and 34.6, respectively. Table 1 reports the top ten articles regarding the annual average number of citations. Their values all exceeded 12.

### 3.2. Qualitative Review Results

Our qualitative review identified two major research streams in the contents of the top ten articles as follows. First, the top ten articles overwhelmingly utilized the technological approach among the three approaches according to Leavitt [12], but they paid little attention to the influence of blockchain technology on the people, structure, and task of healthcare organizations in the integrative perspective of Leavitt’s diamond model [12]. More specifically, most of the top ten articles focused on developing or suggesting technological solutions with blockchain technology to effectively innovate ways of managing medical data in the healthcare domain. For example, Yue, Wang, Jin, Li, and Jiang [13] developed and suggested a blockchain technology-based app named Healthcare Data Gateway to enable patients to effectively manage their medical data through the use of their smartphones. Xia et al. [7] designed and suggested a medical data sharing system using the blockchain called MedShare to effectively manage shared medical data. Guo, Shi, Zhao, and Zheng [5] proposed a safe attribute-based signature (ABS) scheme to securely protect the privacy of patients’ electronic health records with blockchain technology. In line with these studies, the majority of the other studies in Table 1 also adopted the technological approach. However, in the top ten articles, there are few studies that have analyzed the impacts of applying blockchain technology on the people, structure, and task of healthcare organizations under the integrative perspective of Leavitt’s diamond model [12].

Second, the convergence of blockchain technology with cloud technology or IoT is revealed to be salient in the contents of the top ten articles. The majority of the top ten articles concerned new ways of managing medical data by integrating blockchain technology with cloud technology or IoT. With regard to the convergence of blockchain technology with cloud technology, Esposito, De Santis, Tortora, Chang, and Choo [8] analyzed the potential pros and cons of using blockchain technology for healthcare data protection in the environment based on cloud technology. Xia, Sifah, Smahi, Amofa, and Zhang [14] suggested a blockchain-based data sharing (BBDS) system for effectively managing electronic medical data in the context of cloud technology. The blockchain cloud composes one of the three layers essential for the Healthcare Data Gateway suggested by Yue, Wang, Jin, Li, and Jiang [13]. The MeDShare suggested by Xia et al. [7] is a system for medical data sharing to control shared data in the medical data repositories with cloud technology. With regard to the convergence of blockchain technology with IoT, Dwivedi, Srivastava, Dhar, and Singh [9] suggested a new blockchain-based system architecture to solve issues of safety and privacy in medical data transfer through IoT healthcare devices for remote patient monitoring. Griggs et al. [10] developed and proposed a healthcare blockchain system for automated real-time patient monitoring through IoT healthcare devices. The contents of more than five of the top ten articles are based on the technology convergence of blockchain technology with cloud technology or IoT.

## 4. Discussion

Our review results can provide useful implications for future research regarding blockchain technology in the healthcare domain as follows.

First, it is desirable for future studies to pay more attention to the use of the people approach or the structural approach. Our qualitative review results point out that most of the top ten articles adopted the technological approach by concentrating on blockchain-based solutions to current issues in managing medical data without analyzing the impacts of blockchain technology on the people, structure, and task of healthcare organizations in the integrative perspective of Leavitt’s diamond model [12]. As emphasized by Leavitt [12], the technology is a major factor that can transform healthcare organizations, but the inter-relationships between people, technology, structure, and task can ultimately determine the success of blockchain-based solutions in managing medical data in healthcare organizations. No matter how effective the solutions that blockchain technology may provide to healthcare organizations are, the solutions can hardly succeed without considering the harmony of the blockchain-based solutions with the people, structure, and task of healthcare organizations [12]. Therefore, the scope of the major research streams—which mainly focus on the technological approach—can be widened by illuminating a way to ensure harmony by adopting the people approach or the structural approach in future studies.

Second, it is worthwhile to pay special attention to the technology convergence of blockchain technology with cloud technology or IoT in the contents of the top ten articles, as revealed by this review. The majority of the top ten articles suggested blockchain-based solutions with the convergence of blockchain technology with cloud technology or IoT. Blockchain technology has both strengths and limitations [1], facing potential challenges which must be overcome for successfully managing medical data in the healthcare domain [8]. Therefore, the major research streams in blockchain technology in healthcare can be deepened by illuminating new ways of complementing its limitations with the strengths of other technologies through technology convergence.

Third, there is a high demand for an interdisciplinary approach of future studies on blockchain technology in healthcare. It would be effective for future studies to adopt an interdisciplinary approach, rather than a monodisciplinary approach, to provide innovative ways of managing medical data for healthcare organizations. Various views from multiple experts of not only healthcare but also of IT, human psychology, organizational structure, and task are necessary to more accurately analyze the influences of blockchain technology on the people, structure, and task of healthcare organizations and more effectively create blockchain-based solutions for issues of managing medical data.

## 5. Conclusions

In the current study, we illuminated the main research streams through a quantitative and qualitative review, providing implications for future research on blockchain technology.

The quantitative review identified five research streams. First, the number of articles about blockchain technology in healthcare has dramatically increased since 2016, with a compound annual growth rate of 254.4%. Second, English is the most dominant language used in the articles. Third, the USA and China are the top two countries of origin of the articles, representing the overwhelming portions. Fourth, the IEEE Access, Journal of Medical Systems, Journal of Medical Internet Research, Applied Sciences Basel, and Sensors are the top five journals in terms of publication. Fifth, the articles showed an L-shaped distribution in terms of their annual average numbers of citations.

The qualitative review revealed two research streams. First, most of the top 10 articles ranked by their annual average numbers of citations concentrated on developing or proposing new technological solutions using blockchain technology to effectively revolutionize the current ways of managing data in the healthcare domain. Second, the majority of the top 10 studies pursued the convergence of blockchain technology with cloud technology or IoT.

This study provides three implications for future research on blockchain technology in healthcare based on the quantitative and qualitative review. First, it is desirable for future studies to pay more attention to the use of the people approach or the structural approach. The scope of the major research streams mainly focusing on the technological approach can be widened by illuminating a way to ensure harmony by adopting the people approach or the structural approach in future studies. Second, it is worthwhile for future studies to pay special attention to the technology convergence of blockchain technology with cloud technology or IoT. The major research streams in blockchain technology in healthcare can be deepened by illuminating new ways of complementing its limitations with the strengths of other technologies through technology convergence. Third, there is a high demand for future studies to adopt an interdisciplinary approach for blockchain technology in healthcare. It would be effective for future studies to adopt an interdisciplinary approach, rather than a monodisciplinary approach, to provide innovative ways of managing medical data for healthcare organizations.

Our review has limitations that must be overcome in future review papers on blockchain technology in healthcare. It would be desirable to review studies on applying blockchain technology to more specific domains of healthcare. It would be valuable to analyze the contents of more than the top ten articles ranked by the annual average numbers of citations.

## Figures and Tables

**Table 1:** Top ten articles in terms of the annual average numbers of citations.

Rank	Article	Annual Average Numbers of Citations
1	Yue, Wang, Jin, Li, and Jiang [13]	34.6
2	Xia et al. [7]	26.8
3	Esposito, De Santis, Tortora, Chang, and Choo [8]	19.0
4	Guo, Shi, Zhao, & Zheng [5]	16.3
5	Dagher, Mohler, Milojkovic, and Marella [4]	16.0
6	Xia, Sifah, Smahi, Amofa, and Zhang [14]	15.3
7	Dwivedi, Srivastava, Dhar, and Singh [9]	15.0
8	Hussein et al. [15]	13.7
9	Zhang, White, Schmidt, Lenz, and Rosenbloom [16]	12.7
10	Griggs et al. [10]	12.3

## Data Availability

The data presented in this study are available on request from the corresponding author.

## Appendix A

**Table A1:** Distribution of articles by country of origin.

Rank	Country	Frequency	Portion (%)
1	USA	41	30.4
2	China	40	29.6
3	England	11	8.1
3	South Korea	11	8.1
5	India	10	7.4
6	Saudi Arabia	8	5.9
7	Australia	6	4.4
7	Canada	6	4.4
7	Spain	6	4.4
10	Japan	5	3.7
10	Malaysia	5	3.7
10	Taiwan	5	3.7
13	Pakistan	4	3.0
14	Brazil	3	2.2
14	Germany	3	2.2
14	Iraq	3	2.2
14	Italy	3	2.2
14	Norway	3	2.2
19	France	2	1.5
19	Greece	2	1.5
19	Netherlands	2	1.5
19	New Zealand	2	1.5
19	Poland	2	1.5
19	Portugal	2	1.5
19	United Arab Emirates	2	1.5
26	Austria	1	0.7
26	Bangladesh	1	0.7
26	Belgium	1	0.7
26	Bulgaria	1	0.7
26	Colombia	1	0.7
26	Croatia	1	0.7
26	Denmark	1	0.7
26	Ecuador	1	0.7
26	Jordan	1	0.7
26	Libya	1	0.7
26	North Ireland	1	0.7
26	Palestine	1	0.7
26	Philippines	1	0.7
26	Qatar	1	0.7
26	Scotland	1	0.7
26	Thailand	1	0.7
26	Wales	1	0.7

## Appendix B

**Table A2:** Distribution of articles in journal.

Rank	Journal	Frequency	Portion (%)
1	IEEE Access	23	17.0
2	Journal of Medical Systems	16	11.9
3	Journal of Medical Internet Research	14	10.4
4	Applied Sciences Basel	5	3.7
4	Sensors	5	3.7
6	Computational and Structural Biotechnology Journal	3	2.2
6	Electronics	3	2.2
6	Future Generation Computer Systems The International Journal of Escience	3	2.2
6	Wireless Communications Mobile Computing	3	2.2
10	Healthcare Informatics Research	2	1.5
10	Information	2	1.5
10	International Journal of Advanced Computer Science and Applications	2	1.5
10	International Journal of Distributed Sensor Networks	2	1.5
10	Internet Technology Letters	2	1.5
10	Journal of Digital Imaging	2	1.5
10	Journal of The American Medical Informatics Association	2	1.5
10	Pneumologe	2	1.5
18	3c Tecnologia	1	0.7
18	Academic Medicine	1	0.7
18	Applied Health Economics and Health Policy	1	0.7
18	Australasian Journal of Information Systems	1	0.7
18	Big Data Society	1	0.7
18	Bmc Medicine	1	0.7
18	Bmj Global Health	1	0.7
18	Business Process Management Journal	1	0.7
18	Clinics in Dermatology	1	0.7
18	Cognitive Systems Research	1	0.7
18	Computers	1	0.7
18	Concurrency and Computation Practice Experience	1	0.7
18	Cryptologia	1	0.7
18	Future Internet	1	0.7
18	Health Informatics Journal	1	0.7
18	IBM Journal of Research and Development	1	0.7
18	IEEE Cloud Computing	1	0.7
18	IEEE Consumer Electronics Magazine	1	0.7
18	IEEE Internet of Things Journal	1	0.7
18	IEEE Network	1	0.7
18	IEEE Transactions on Computational Social Systems	1	0.7
18	Industrial Management Data Systems	1	0.7
18	Information Sciences	1	0.7
18	International Journal of Cardiology	1	0.7
18	International Journal of Engineering Business Management	1	0.7
18	International Journal of Environmental Research and Public Health	1	0.7
18	International Journal of Healthcare Information Systems and Informatics	1	0.7
18	International Journal of Healthcare Management	1	0.7
18	International journal of Networked and Distributed Computing	1	0.7
18	JMIR Mhealth and Uhealth	1	0.7
18	JMIR Research Protocols	1	0.7
18	Journal of Biomedical Informatics	1	0.7
18	Journal of Information Assurance and Security	1	0.7
18	Journal of Information Processing Systems	1	0.7
18	Journal of Information Security and Applications	1	0.7
18	Journal of the American College of Radiology	1	0.7
18	Nature Communications	1	0.7
18	Neural Computing Applications	1	0.7
18	Parkinsonism Related Disorders	1	0.7
18	Procesamiento Del Lenguaje Natural	1	0.7
18	Security and Communication Networks	1	0.7
18	Sustainable Cities and Society	1	0.7
18	Technology Innovation Management Review	1	0.7
18	Unfallchirurg	1	0.7
